# Supplementary material for: Updated Risk Assessment of Cannabidiol in Foods Based on Benchmark Dose–Response Modeling
Source: Molecules. 2024 Oct 7;29(19):4733. doi: 10.3390/molecules29194733 (PMC11477611; doi:10.3390/molecules29194733)
Supplement: Supplementary file 1 [file molecules-29-04733-s001.zip › dziwenka-et-al-90-day-oral-toxicity.pdf]

Dziwenka et al. 90 day oral Toxicity

Elixinol hemp extract in rats

Report generated: 2024-Jun-12 10:06 UTC

BMDS version: 3.3.0 (2023.03.1)

BMDS online version: d5f505c8 (2023-03-14)

Session Results

Input Dataset

Dose (mg/kg bw/day)	N	Mean (g)	Std. Dev. (g)
0	15	13.289	2.238
18.95	15	14.742	2.425
33.16	15	14.795	1.418
56.84	15	16.503	2.507

Input Settings

Setting	Value
**BMR**	1.0 Standard Deviation
**Distribution**	Normal + Constant variance
**Modeling Direction**	Up (↑)
**Maximum Polynomial Degree**	1
**Confidence Level**	0.95
**Tail Probability**	0.01

Frequentist Summary

Model	BMDL	BMD	BMDU	*P*-Value	AIC	Scaled Residual for Dose Group near BMD	Scaled Residual for Control Dose Group	Recommendation and Notes
ExponentialM3[a]	29.822	41.143	69.648	0.644	267.084	-0.57	-0.23	**Recommended - Lowest AIC**
ExponentialM5	30.891	39.875	55.968	0.348	269.085	-0.643	-0.159	**Viable**
Hill	17.713	38.79	1108.726	-	271.141	-0.712	-0.111	**Questionable** Zero degrees of freedom; saturated model
Power	27.781	39.884	70.531	0.644	267.085	-0.643	-0.159	**Viable**
Linear	27.78	39.884	70.531	0.644	267.085	-0.643	-0.159	**Viable**

[a] Recommended best-fitting model

Selected Model

ExponentialM3

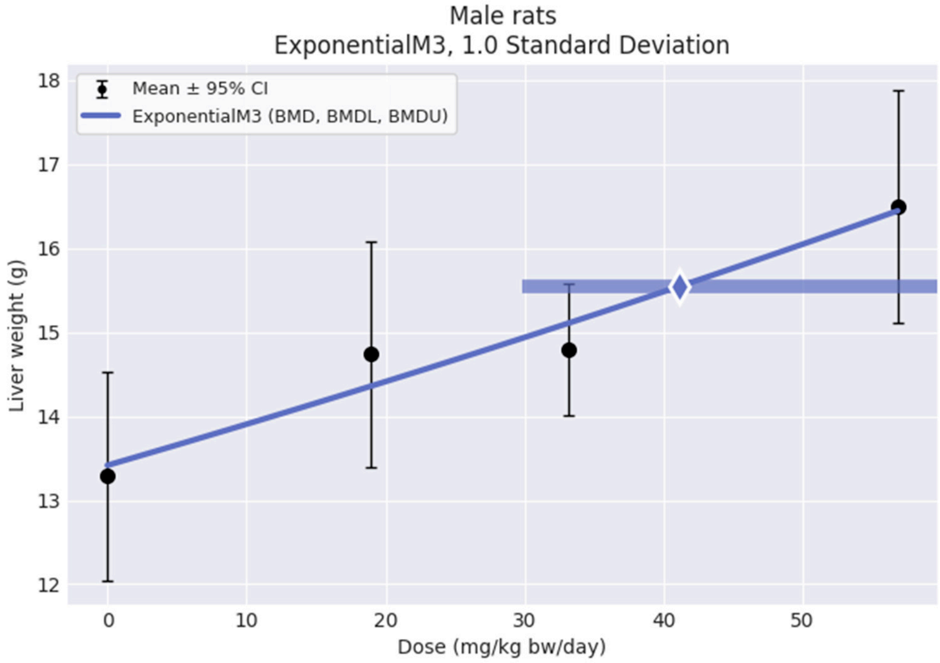

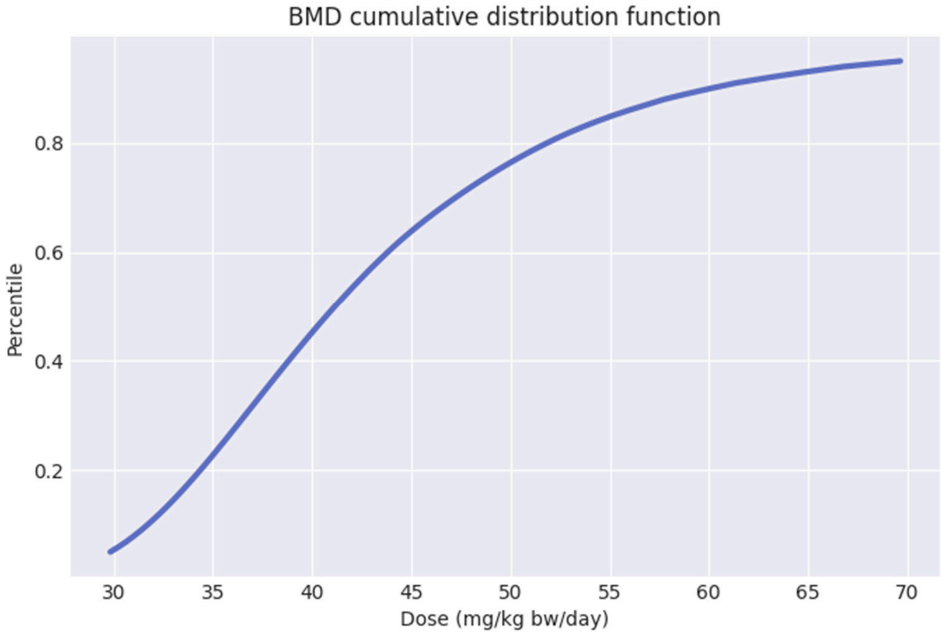


```
    ExponentialM3
══════════════════

Input Summary:
```

BMR	1.0 Standard Deviation
Distribution	Normal + Constant variance
Modeling Direction	Up (↑)
Confidence Level	0.95
Tail Probability	0.01
Modeling Approach	Frequentist restricted

Parameter Settings:

Parameter	Initial	Min	Max
a	0	0	100
b	0	0	100
c	0	0	20
d	0	1	18
log-alpha	0	-18	18

Summary:

BMD	41.1427
BMDL	29.822
BMDU	69.6478
AIC	267.084
Log Likelihood	130.542
P-Value	0.643913
Model DOF	2

Model Parameters:

Variable	Estimate	Bounded	Std Error	Lower CI	Upper CI
a	13.4156	no	0.438339	12.5565	14.2747
b	0.00358378	no	0.000887584	0.00184415	0.00532341
d	1	yes	NA	NA	NA
log-alpha	1.51352	no	0.182578	1.15567	1.87136

Goodness of Fit:

Dose	Size	Observed Mean	Calculated Mean	Estimated Mean	Scaled Residual
0	15	13.289	13.289	13.4156	-0.230087
18.95	15	14.742	14.742	14.3584	0.697131
33.16	15	14.795	14.795	15.1085	-0.569683
56.84	15	16.503	16.503	16.4466	0.102401

Dose	Size	Observed SD	Calculated SD	Estimated SD
0	15	2.238	2.238	2.13136
18.95	15	2.425	2.425	2.13136
33.16	15	1.418	1.418	2.13136
56.84	15	2.507	2.507	2.13136

Likelihoods of Interest:

Model	Log Likelihood	# Params	AIC
A1	-130.102	5	270.203
A2	-127.463	8	270.926
A3	-130.102	5	270.203
fitted	-130.542	3	267.084
reduced	-137.728	2	279.455

Tests of Interest:

Name	Loglikelihood Ratio	Test DOF	P-Value
Test 1	20.5298	6	0.00222764
Test 2	5.27774	3	0.152553
Test 3	5.27774	3	0.152553
Test 4	0.880384	2	0.643913
