# Supplementary material for: Updated Risk Assessment of Cannabidiol in Foods Based on Benchmark Dose–Response Modeling
Source: Molecules. 2024 Oct 7;29(19):4733. doi: 10.3390/molecules29194733 (PMC11477611; doi:10.3390/molecules29194733)
Supplement: Supplementary file 1 [file molecules-29-04733-s001.zip › henderson-et-al-90-day-oral-toxicity-study.pdf]

Henderson et al. 90-Day oral toxicity study

CBD-isolat tested in rats

Report generated: 2024-Jun-12 10:06 UTC

BMDS version: 3.3.0 (2023.03.1)

BMDS online version: d5f505c8 (2023-03-14)

Session Results

Input Dataset

Dose (mg/kg bw/day)	N	Mean (g)	Std. Dev. (g)
0	10	8.52	0.84
50	10	8.71	1.13
80	10	10.64	1.22
120	10	12.13	1.16
140	10	12.98	2.05

Input Settings

Setting	Value
**BMR**	1.0 Standard Deviation
**Distribution**	Normal + Constant variance
**Modeling Direction**	Up (↑)
**Maximum Polynomial Degree**	1
**Confidence Level**	0.95
**Tail Probability**	0.01

Frequentist Summary

Model	BMDL	BMD	BMDU	*P*-Value	AIC	Scaled Residual for Dose Group near BMD	Scaled Residual for Control Dose Group	Recommendation and Notes
ExponentialM3	41.505	63.441	88.011	0.2	177.336	-1.341	0.407	**Viable**<br>Control stdev. fit greater than 1.5
ExponentialM5	48.594	66.644	84.997	0.239	177.504	0.615	0.317	**Viable**<br>Control stdev. fit greater than 1.5
Hill	50.351	67.497	83.624	0.338	177.04	0.368	0.231	**Viable**<br>Control stdev. fit greater than 1.5
Power[a]	42.994	63.971	87.538	0.242	176.957	-1.248	0.402	**Recommended - Lowest AIC**<br>Control stdev. fit greater than 1.5
Linear	32.037	40.087	53.247	0.061	179.503	-2.139	1.367	**Questionable**<br>Goodness of fit p-value less than 0.1<br>Control stdev. fit greater than 1.5<br>Abs(Residual of interest) greater than 2.0

[a] Recommended best-fitting model

Selected Model
Power

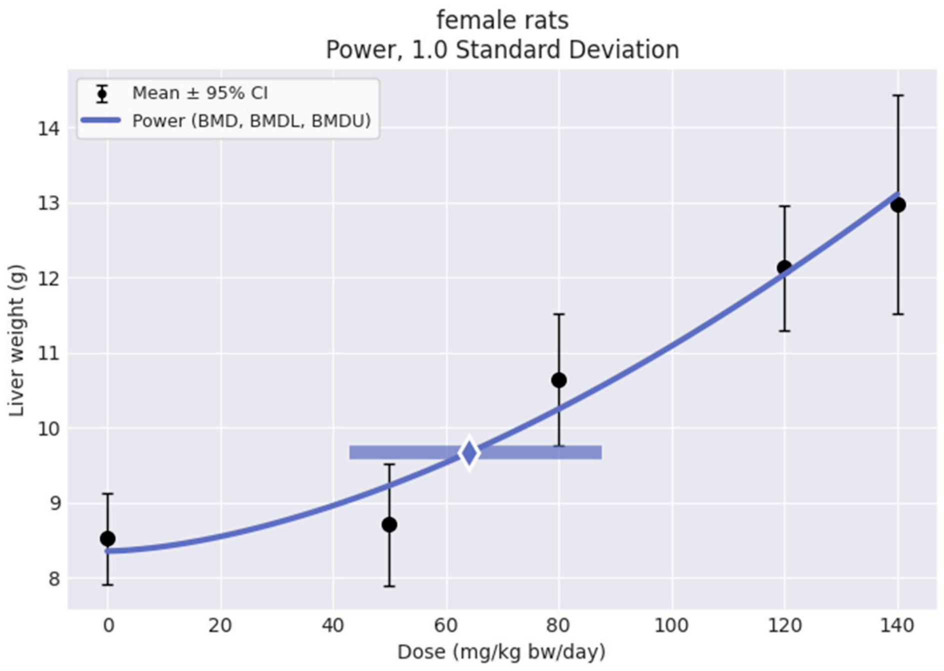

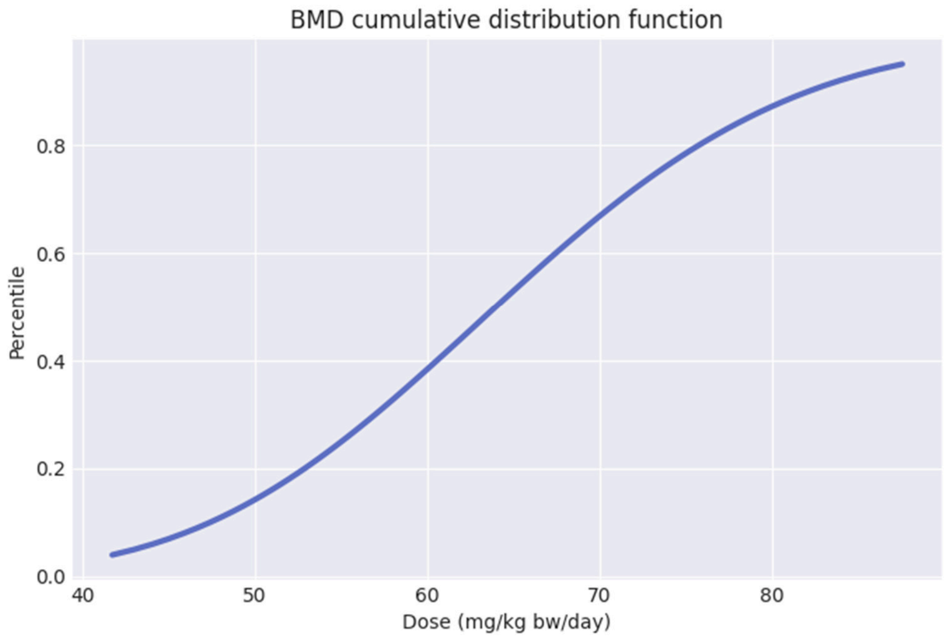


```
        Power
════════════════════

Input Summary:
```

BMR	1.0 Standard Deviation
Distribution	Normal + Constant variance
Modeling Direction	Up (↑)
Confidence Level	0.95
Tail Probability	0.01
Modeling Approach	Frequentist restricted

Parameter Settings:

Parameter	Initial	Min	Max
g	0	-100	100
v	0	-100	100
n	0	1	18
alpha	0	-18	18

Summary:

BMD	63.9705
BMDL	42.9939
BMDU	87.5384
AIC	176.957
Log Likelihood	84.4786
P-Value	0.242127
Model DOF	2

Model Parameters:

Variable	Estimate	Bounded	Std Error	Lower CI	Upper CI
g	8.35348	no	0.382399	7.60399	9.10297
v	0.00140047	no	0.00015036	0.00110577	0.00169517
n	1.64523	no	0.382041	0.896449	2.39402
alpha	1.71818	no	0.590439	0.560942	2.87542

Goodness of Fit:

Dose	Size	Observed Mean	Calculated Mean	Estimated Mean	Scaled Residual
0	10	8.52	8.52	8.35348	0.40172
50	10	8.71	8.71	9.22742	-1.24826
80	10	10.64	10.64	10.2471	0.947747
120	10	12.13	12.13	12.0434	0.208956
140	10	12.98	12.98	13.1086	-0.310163

Dose	Size	Observed SD	Calculated SD	Estimated SD
0	10	0.84	0.84	1.31079
50	10	1.13	1.13	1.31079
80	10	1.22	1.22	1.31079
120	10	1.16	1.16	1.31079
140	10	2.05	2.05	1.31079

Likelihoods of Interest:

Model	Log Likelihood	# Params	AIC
A1	-83.0603	6	178.121
A2	-78.4427	10	176.885
A3	-83.0603	6	178.121
fitted	-84.4786	4	176.957
reduced	-110.186	2	224.371

Tests of Interest:

Name	Loglikelihood Ratio	Test DOF	P-Value
Test 1	63.486	8	9.60795e-11
Test 2	9.23528	4	0.0554803
Test 3	9.23528	4	0.0554803
Test 4	2.83659	2	0.242127
