# Supplementary material for: Updated Risk Assessment of Cannabidiol in Foods Based on Benchmark Dose–Response Modeling
Source: Molecules. 2024 Oct 7;29(19):4733. doi: 10.3390/molecules29194733 (PMC11477611; doi:10.3390/molecules29194733)
Supplement: Supplementary file 1 [file molecules-29-04733-s001.zip › tallon-child-90-day-oral-toxicity.pdf]

Tallon & Child 90 day oral Toxicity

CBD isolate on rats

Report generated: 2024-Jun-12 10:06 UTC

BMDS version: 3.3.0 (2023.03.1)

BMDS online version: d5f505c8 (2023-03-14)

Session Results

Input Dataset

Dose (mg/kg bw/day)	N	Mean (g)	Std. Dev. (g)
0	15	9.12	1.17
30	15	9.64	1.45
115.13	15	10.47	0.84
230.25	15	12.11	1.11
460.5	15	15.01	1.47

Input Settings

Setting	Value
**BMR**	1.0 Standard Deviation
**Distribution**	Normal + Constant variance
**Modeling Direction**	Up (↑)
**Maximum Polynomial Degree**	1
**Confidence Level**	0.95
**Tail Probability**	0.01

Frequentist Summary

Model	BMDL	BMD	BMDU	*P*-Value	AIC	Scaled Residual for Dose Group near BMD	Scaled Residual for Control Dose Group	Recommendation and Notes
ExponentialM3	99.76	114.999	150.792	0.762	245.936	-0.094	-0.581	**Viable**
ExponentialM5	79.548	97.062	143.197	0.557	249.117	-0.38	-0.157	**Viable**
Hill	68.549	101.243	150.038	0.546	249.137	-0.309	-0.24	**Viable**
Power	79.801	96.963	146.771	0.842	247.117	-0.382	-0.155	**Viable**
Linear[a]	79.778	93.661	112.873	0.948	245.136	-0.448	-0.075	**Recommended - Lowest AIC**

[a] Recommended best-fitting model

Selected Model
Linear

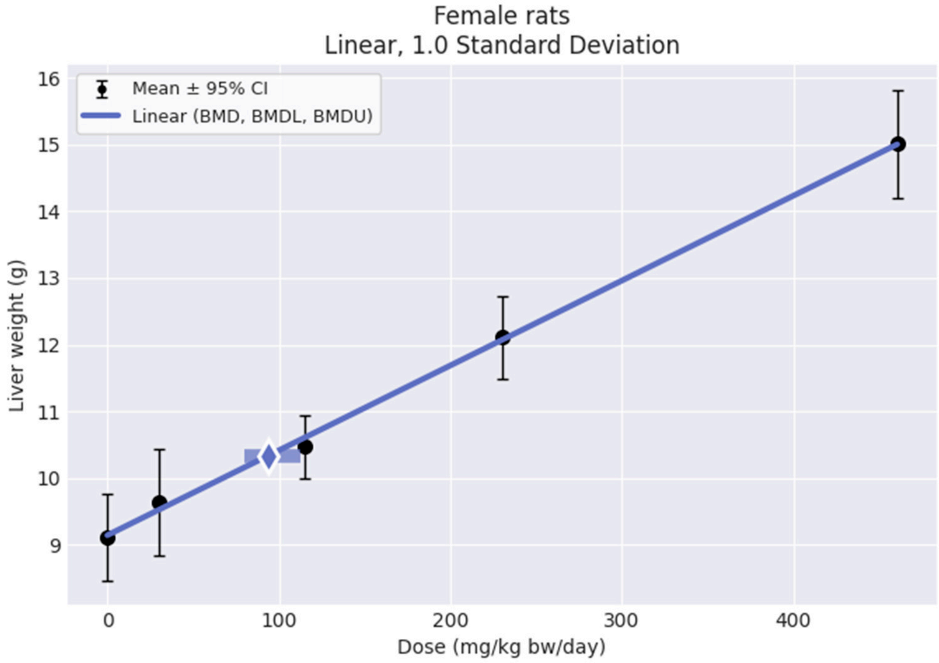

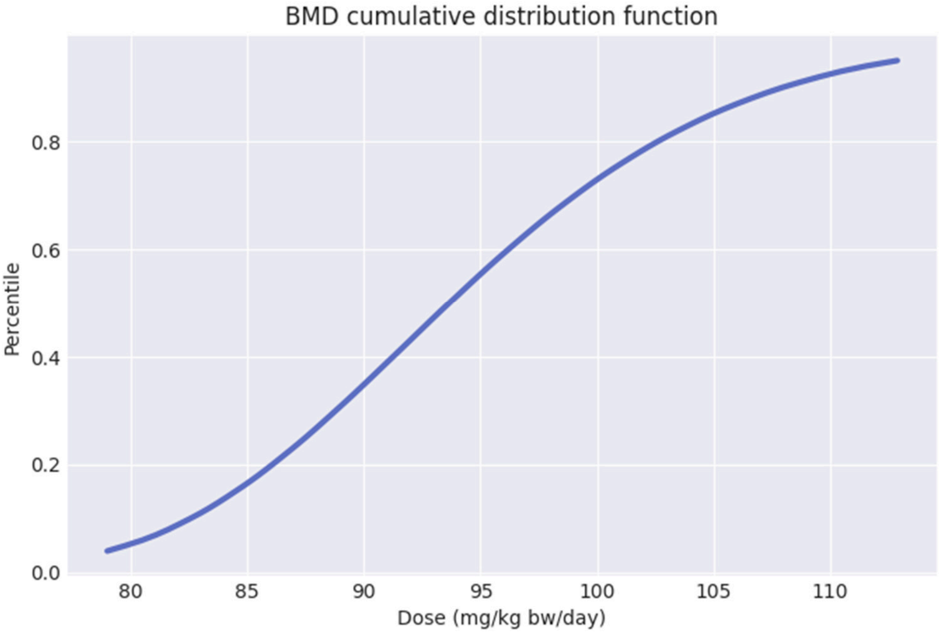


```
        Linear
═══════════════════════

Input Summary:
```

BMR	1.0 Standard Deviation
Distribution	Normal + Constant variance
Modeling Direction	Up (↑)
Confidence Level	0.95
Tail Probability	0.01
Modeling Approach	Frequentist unrestricted

Parameter Settings:

Parameter	Initial	Min	Max
g	0	-1e+06	1e+06
b1	0	-1e+06	1e+06
alpha	0	-1e+06	1e+06

Summary:

BMD	93.6613
BMDL	79.7779
BMDU	112.873
AIC	245.136
Log Likelihood	119.568
P-Value	0.947758
Model DOF	3

Model Parameters:

Variable	Estimate	Bounded	Std Error	Lower CI	Upper CI
g	9.1431	no	0.194675	8.76155	9.52466
b1	0.0127225	no	0.000823787	0.0111079	0.0143371
alpha	1.41992	no	0.32929	0.774528	2.06532

Goodness of Fit:

Dose	Size	Observed Mean	Calculated Mean	Estimated Mean	Scaled Residual
0	15	9.12	9.12	9.1431	-0.0750918
30	15	9.64	9.64	9.52478	0.374495
115.13	15	10.47	10.47	10.6078	-0.448026
230.25	15	12.11	12.11	12.0725	0.122017
460.5	15	15.01	15.01	15.0018	0.0266059

Dose	Size	Observed SD	Calculated SD	Estimated SD
0	15	1.17	1.17	1.19161
30	15	1.45	1.45	1.19161
115.13	15	0.84	0.84	1.19161
230.25	15	1.11	1.11	1.19161
460.5	15	1.47	1.47	1.19161

Likelihoods of Interest:

Model	Log Likelihood	# Params	AIC
A1	-119.386	6	250.773
A2	-116.491	10	252.981
A3	-119.386	6	250.773
fitted	-119.568	3	245.136
reduced	-173.207	2	350.413

Tests of Interest:

Name	Loglikelihood Ratio	Test DOF	P-Value
Test 1	113.432	8	0
Test 2	5.79155	4	0.215266
Test 3	5.79155	4	0.215266
Test 4	0.363087	3	0.947758
